# Preventive Impact of Long-Term Ingestion of Chestnut Honey on Glucose Disorders and Neurodegeneration in Obese Mice

**DOI:** 10.3390/nu14040756

**Published:** 2022-02-11

**Authors:** Simona Terzo, Pasquale Calvi, Domenico Nuzzo, Pasquale Picone, Giacoma Galizzi, Luca Caruana, Marta Di Carlo, Laura Lentini, Roberto Puleio, Flavia Mulè, Antonella Amato

**Affiliations:** 1 Dipartimento di Scienze e Tecnologie Biologiche, Chimiche e Farmaceutiche (STEBICEF), Università degli Studi di Palermo, 90128 Palermo, Italy; simona.terzo01@unipa.it (S.T.); pasquale.calvi@unipa.it (P.C.); domenico.nuzzo@cnr.it (D.N.); pasquale.picone@cnr.it (P.P.); laura.lentini@unipa.it (L.L.); flavia.mule@unipa.it (F.M.); 2 Dipartmento di Biomedicina, Neuroscienze e Diagnostica Avanzata (Bi.N.D.), Università degli Studi di Palermo, 90127 Palermo, Italy; 3 Istituto per la Ricerca e l’Innovazione Biomedica (IRIB), CNR, via U. La Malfa 153, 90146 Palermo, Italy; giacoma.galizzi@irib.cnr.it (G.G.); luca.caruana@irib.cnr.it (L.C.); marta.dicarlo@irib.cnr.it (M.D.C.); 4 Istituto Zooprofilattico Sperimentale della Sicilia “Adelmo Mirri”, 90129 Palermo, Italy; roberto.puleioizs@gmail.com

**Keywords:** honey, obesity, neurodegeneration, insulin resistance, HFD mice

## Abstract

The purpose of the present study was to evaluate the impact of long-term honey ingestion on metabolic disorders and neurodegeneration in mice fed a high-fat diet (HFD). Three groups of mice were fed with a standard diet (STD), HFD or HFD supplemented with honey (HFD-H) for 16 weeks. Biochemical, histological, Western blotting, RT-PCR and Profiler PCR array were performed to assess metabolic parameters, peripheral and central insulin resistance and neurodegeneration. Daily honey intake prevented the HFD-induced glucose dysmetabolism. In fact, it reduced plasma fasting glucose, insulin and leptin concentrations and increased adiponectin levels. It improved glucose tolerance, insulin sensitivity and HOMA index without affecting plasma lipid concentration. HFD mice showed a significantly higher number of apoptotic nuclei in the superficial and deep cerebral cortex, upregulation of *Fas-L*, *Bim* and *P27* (neuronal pro-apoptotic markers) and downregulation of *Bcl-2* and *BDNF* (anti-apoptotic factors) in comparison with STD- and HFD-H mice, providing evidence for honey neuroprotective effects. PCR-array analysis showed that long-term honey intake increased the expression of genes involved in insulin sensitivity and decreased genes involved in neuroinflammation or lipogenesis, suggesting improvement of central insulin resistance. The expressions of p-AKT and p-GSK3 in HFD-H mice, which were decreased and increased, respectively, in HFD mouse brain, index of central insulin resistance, were similar to STD animals supporting the ability of regular honey intake to protect brain neurons from insulin resistance. In conclusion, the present results provide evidence for the beneficial preventative impact of regular honey ingestion on neuronal damage caused by HFD.

## 1. Introduction

Neurodegenerative diseases represent a relevant health problem. Indeed, the World Health Organization has stated that they will become the worldwide second reason of death by the middle of the 21st century after cancer [1]. They include diverse pathological conditions, among which Alzheimer’s and Parkinson’s diseases are the most prevalent ones [2]. Neurodegeneration is characterized by loss of specific neurons, leading to progressive dysfunctions of the central nervous system. The etiology of neurodegenerative diseases is multifactorial, and it involves mainly aging as well as genetics and environmental factors [3,4]. Currently, it is well accepted that unhealthy lifestyles and dietary habits increase the risk of developing neurodegenerative diseases [5]. Indeed, obesity and type 2 diabetes mellitus accelerate the neurodegenerative process, neuroinflammation and oxidative stress accountable for the progressive cognitive decline [6,7]. Actually, the various components of metabolic syndrome (dyslipidemia, obesity, hyperglycemia, insulin resistance and hypertension) cause wide-ranging effects, some of which affect the central nervous system (CNS), and they may result in neurodegenerative diseases [8]. The excessive long-term ingestion of a high-fat diet (HFD) alters body homeostasis, and it participates in the pathogenesis of neurodegeneration [9,10,11], which is responsible for impairment of cognitive functions in rodents [11]. 

No effective protective or therapeutic approaches are currently available against obesity-induced brain injury and irreversible loss of neurons. In recent years, various in vitro studies or experiments on preclinical animal models have demonstrated the use of bioactive phytochemicals, such as resveratrol, curcumin, quercetin or functional food, which is able to limit brain damage through their antioxidant and anti-inflammatory properties [12,13,14,15,16]. 

Honey has been traditionally considered as a supporting factor in medical treatment since ancient times. The health-promoting characteristics are due to the presence of molecules with recognized antioxidants and anti-inflammatory properties [17]. Although honey is speculated to be a potential agent in preventing and reversing metabolic syndrome by reducing obesity, hyperglycemia, dyslipidemia and hypertension [18,19,20,21,22], the consequences of honey chronic ingestion on glucose homeostasis are still debated, and the impact on brain health conditions remains poorly known. Therefore, the present study was undertaken in order to evaluate the effects of regular honey intake on HFD-induced neurodegeneration in an animal model. In addition, we examined if an improvement in glucose dysmetabolism might be directly involved in the eventual neuroprotective effect.

## 2. Materials and Methods

### 2.1. Animals and Diets

The animal procedures for the care and use of laboratory animals were performed accordingly in conformity with the Italian legislative decree n. 26/2014 and the European Directive 2010/63/UE and were authorized by the Ministry of Health (Rome, Italy; Authorization n. 891/2018-PR). 

Four-week-old male C57BL/6J mice, purchased from Harlan Laboratories (San Pietro al Natisone Udine, Italy) were housed (two animals per cage) in a room with a controlled environment of temperature (22–24 °C), and relative humidity (55 ± 5%) with 12 h light/dark and with free access to food and water ad libitum.

After acclimatization (1 week), the animals were weighed and divided into separated three groups: (A) STD group (STD, *n* = 8) fed a standard diet consisting of 70% of energy as carbohydrate, 20% protein and 10% fat (code 4RF25, Mucedola, Milan, Italy) (Table S1) (Supplementary Data); (B) High-fat diet group (HFD, *n* = 8), fed HFD (PF4215, Mucedola, Milan, Italy) that, as previously described [23], supplied 60% of energy as fat, 20% protein and 20% (Table S1) (Supplementary Data); and (C) Mice fed an HFD supplemented with Sicilian black bee chestnut honey (HFD-H, *n* = 8). HFD-H was obtained by substituting part of the HFD with honey in order to have the same caloric value of HFD. The honey amount was chosen on the basis of previous reports [24], and it corresponds to 45 mg honey ingested/day/mouse. The honey composition is reported in Table S2 (Supplementary Data). The honey amount was chosen on the basis of previous reports [24], and it corresponds to 45 mg honey ingested/day/mouse. Bodyweight and food intake were detected weekly throughout the study.

At the end of the study period, metabolic parameters were analyzed, then the animals were weighed and sacrificed by cervical dislocation. Blood was immediately drawn by cardiac puncture, and plasma was recovered after centrifugation at 3000 rpm at 4 °C for 15 min and stored at −80 °C until analysis. The aorta was cannulated and perfused with Dulbecco’s buffered solution containing 2 mM EDTA and incision of the right atrial allowed blood outflow. Brains were explanted, weighed and processed for subsequent analysis.

### 2.2. Metabolic Parameters

Plasma triglyceride, total cholesterol, HDL and LDL concentrations were measured using the ILAB 600 Analyzer (Instrumentation Laboratory, Bedford, MA, USA). Fasting blood glucose concentrations were determined by a glucometer (GlucoMen LX meter, Menarini, Florence, Italy). Intraperitoneal glucose tolerance test (IPGTT) and insulin tolerance test (ITT) were carried out in overnight fasting mice. For IPGTT, mice were injected intraperitoneally (i.p.) with glucose (2 g/kg b.w.) (D-glucose, Sigma-Aldrich, Milan, Italy) in 0.9% saline. For ITT, mice were injected i.p. with insulin (0.5 U/kg b.w.) (Insuman Rapid, Sanofi Aventis, Italy) in 0.9% saline. Glucose concentrations were measured at different time intervals (0, 15, 30, 60, 120 min) by tail vein. Plasma insulin was quantified using a mouse ELISA kit (Alpco diagnostics, Salem, NH, USA) according to the manufacturer’s instructions. The HOMA-IR index was calculated as the product of fasting insulin (ng/mL) and fasting glucose (mg/dL) divided by the constant 22.5. 

ELISA kits were used to determine plasma leptin (Life Technologies, Frederick, MD, USA) and adiponectin (Crystal Chem, Elk Grove Village, IL, USA).

### 2.3. Brain Tissue Preparation 

Brains obtained from all the groups of animals were coronally cut into two halves. One part was frozen in liquid nitrogen and stored until the subsequent analysis. The other half brain was fixed in 4% formalin for 24 h, dehydrated by graded ethanol (50%, 70%, 85%, 96% for 5 min), then embedded in paraffin overnight and subsequently sectioned (5 µm thick) using a microtome for histological analysis. 

### 2.4. TUNEL Assay

Terminal deoxynucleotidyl Transferase Biotin-dUTP Nick End Labeling (TUNEL) assay was used to detect apoptotic nuclei in brain sections by using a cell death detection kit (Promefa, Madison, WI, USA) according to the manufacturer’s instructions. The number of apoptotic cells was counted in the cerebral cortex selected fields by two of us blind to ingested diet, and the ratio of apoptotic cells per brain area was calculated. 

### 2.5. Western Blotting and PCR

Total proteins were prepared by resuspending 10 mg of frozen homogenate in lysis buffer (50 mM Tris-HCl, pH 7.4; 150 mM NaCl, 0.5% Triton X-100, 2 mM phenylmethylsulfonyl fluoride, 1 mM DTT, 0.1% SDS) with protease inhibitors (Amersham, Life Science, Les Ulis, France) and phosphatase inhibitor cocktail II (Sigma-Aldrich, Milan, Italy). Then, they were quantified by the Bradford method (Bio-Rad, Segrate, Italy). Protein (50 µg) were resolved by 12% acrylamide gel and transferred onto nitrocellulose filters for Western blotting using anti-GSK-3α/β (D75D3) #5676 (1:1000), anti-phosphoGSK-3β (Tyr216) #44-604G (1:500), anti-AKT #9272 (1:1000), anti-phospho-AKT (S473) (D9E) #4060 (1:500), anti-insulin receptor (E9L5V) #23413 (1:500), anti-Erk (137F5) #4695 (1:1000), anti-phospho-Erk (Thr202/Tyr204) #4370 (1:1000), anti-β-Actin A5441 (1: 10,000). All antibodies were supplied by Cell Signaling Technology (Danvers, MA, USA) except anti-phosphoGSK-3β (Tyr216) supplied by Thermofisher and anti-β-actin by Sigma-Aldrich (St. Louis, MI, USA). The primary antibodies were revealed by using secondary antibodies (anti-mouse or anti-rabbit) labeled with infrared fluorescent dye IR680 (1:10,000; LI-COR). Secondary antibodies were detected by LI-COR^®^ 150 Platform (LI-COR, Lincoln, NE, USA), according to the manufacturer’s instructions. Band intensities were analyzed with the Odyssey^®^ CLx Imaging System (Li-Cor, Lincoln, NE, USA), and expression was adjusted to the β-actin expression. The protein levels were expressed as intensity relative to the control. 

For RT-PCR, RNA was extracted using the RNeasy Plus Mini Kit (Qiagen, Valencia, CA, USA). Two ng of RNA were used to obtain cDNA using High Capacity cDNA Reverse Transcription (Applied Biosystems, Bedford, MA, USA). Gene expression was performed using primers listed in Table 1.

The amplification cycles included denaturation at 95° C for 45 s, annealing for 45 s, and elongation at 72 °C for 45 s. After 35 cycles, the amplification products were separated by electrophoresis, and the gels were stained with 1 mg/mL ethidium bromide and visualized with ultraviolet (UV) light using E-Gel GelCapture (Thermo Fisher Scientific, Monza, Italy). The expression levels of the gene targets, normalized to the endogenous reference (β-actin), were analyzed using E-Gel GelQuant Express Analysis Software (Thermo Fisher Scientific, Monza, Italy).

### 2.6. RT2Profiler PCR Array 

Mouse insulin resistance arrays (Insulin Resistance PCR Array, QIAGEN, Monza, Italy) in 96-well plate format were used to assay gene expression changes in the brain. Samples were prepared from pooled RNA extracted from STD, HFD and HFD-H mouse brains. Samples were added to the reaction plates following the manufacturer’s instructions, and a StepOne Real-Time instrument (Applied Biosystem) was used to perform the array. Analysis was performed using the relative quantification method (2^−ΔΔCT^). 

### 2.7. Statistical Analysis 

The results are presented as mean ± S.E.M. Statistical evaluation was performed by ANOVA, followed by Bonferroni post hoc test using Prism 6.0, GraphPad (San Diego, CA, USA). Results with a *p*-value < 0.05 were considered statistically significant.

## 3. Results

### 3.1. Honey Ingestion and Metabolic Parameters

**Figure 1:**
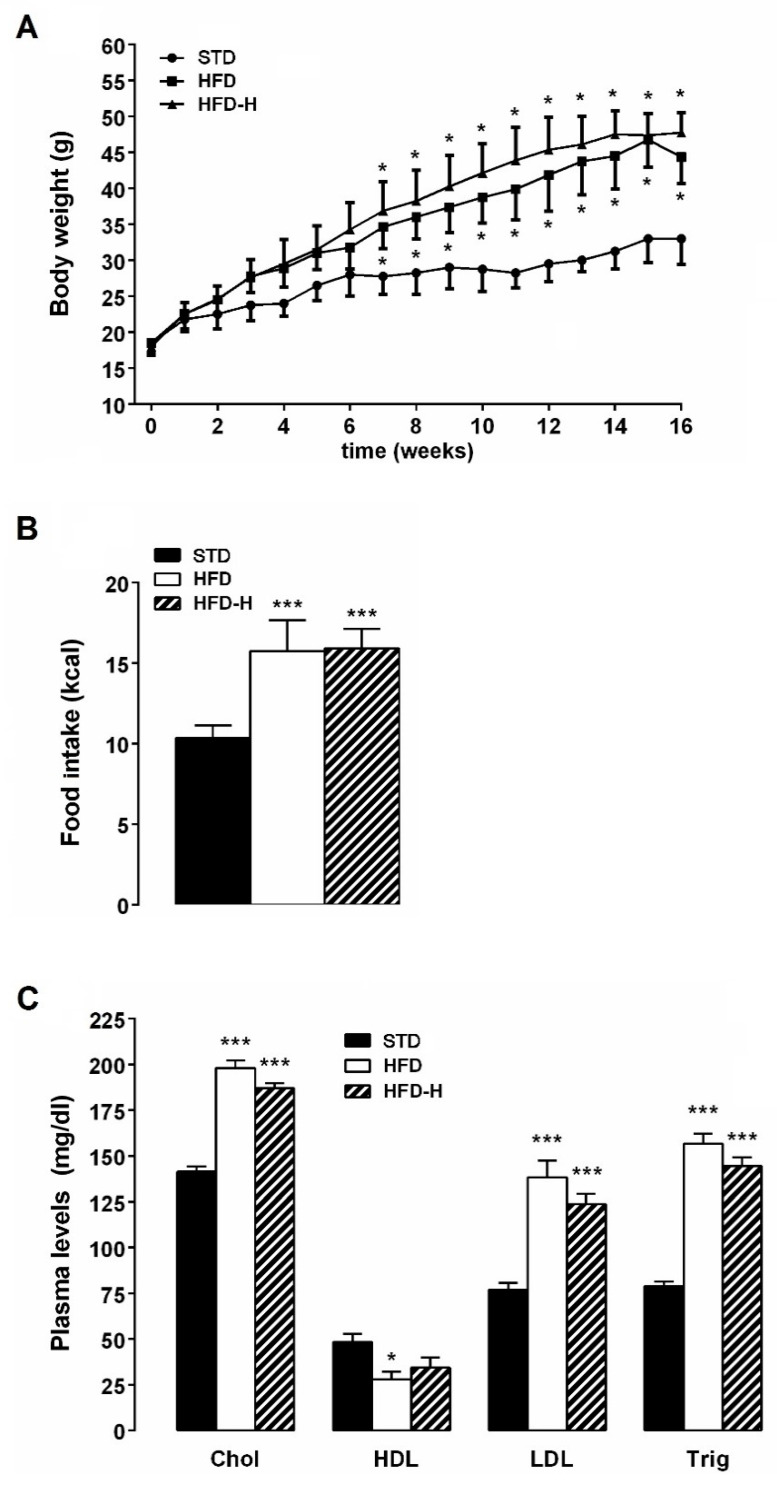
Effects of long-term honey ingestion on metabolic parameters in HFD mice. Honey failed to prevent weight gain and dyslipidemia in HFD mice. (**A**) Bodyweight; (**B**) Daily food intake; (**C**) Plasma concentrations of triglycerides, cholesterol, HDL and LDL in the different groups of mice. Data are mean values ± S.E.M. (*n* = 8/group). * *p* < 0.05, *** *p*< 0.001 vs. STD mice.

All animals gained weight during the experimental period. The bodyweight of HFD and HFD-H mice was significantly higher than STD mice, but no difference was observed when compared HFD-H to HFD. Although the daily caloric intake of HFD-mice was higher than STD mice, it did not result in significantly different in comparison with HFD-H mice (Figure 1A,B). HFD chronic ingestion-induced dyslipidemia, i.e., plasma triglyceride, total cholesterol, low-density lipoprotein (LDL), were increased while levels of high-density lipoproteins (HDL) were reduced in comparison with STD mice (Figure 1C). Honey supplementation failed to prevent the lipid concentration changes associated with HFD consumption (Figure 1C). 

### 3.2. Honey Ingestion Improves Glucose Metabolism in HFD Mice

**Figure 2:**
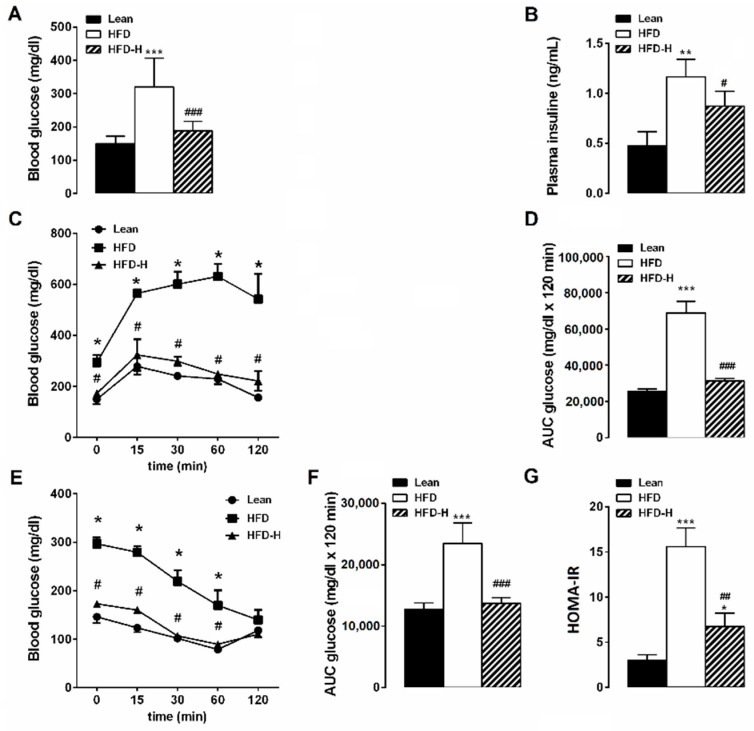
Effects of long-term honey ingestion on glucose metabolism in HFD mice. Honey prevented the glucose dysmetabolism. (**A**) Fasting glucose concentration; (**B**) Fasting insulin concentration; (**C**) Plasma glucose concentration during intraperitoneal glucose tolerance test (IPGTT). (**D**) Area under the curve (AUC) during IPGTT; (**E**) Glucose concentrations during insulin tolerance test (ITT). (**F**) AUC for blood glucose concentrations during ITT; (**G**) HOMA-index calculated as fasting glucose (mg/dL) × fasting insulin (ng/mL)/22.5. Data are mean values ± S.E.M. (*n* = 8 mice/group). * *p* < 0.05, ** *p* < 0.01, *** *p* < 0.001 vs. STD mice; ^#^ *p* < 0.05, ^##^ *p* < 0.01, ^###^ *p* < 0.001 vs. HFD-fed mice.

After 16 weeks on HFD, mice presented hyperglycemia with fasting glucose concentrations 296.6 mg ± 14.8 mg/dL (*n* = 8) significantly higher than STD mice (146.8 ± 9.9 mg/dl, *n* = 8). Honey supplementation improved fasting glucose concentrations. Indeed, glycemia value in HFD-H mice was significantly lower than HFD and similar to STD, suggesting a preventive action against hyperglycemia (Figure 2A). In HFD mice, fasting plasma insulin concentration was three-fold higher compared to STD mice. In HFD-H mice, the increase in plasma insulin caused by HFD was mitigated (Figure 2B). In addition, HFD mice displayed an impaired glycemic response following an intraperitoneal glucose load. Glucose tolerance test demonstrated that basal glycemia increased to a maximum after 15 min of glucose intraperitoneal injection in HFD and STD groups, but this maximum was significantly higher and maintained more elevated in HFD mice than in STD mice (Figure 2C). HFD-H mice showed improved glycemic control as indicated by the reduction in blood glucose levels during the i.p. glucose tolerance test (Figure 2C). The HFD group showed the Glucose area under the curve (AUC) significantly increased compared to STD or HFD-H groups (Figure 2D). The glucose clearance rate in response to exogenous insulin was evaluated in the different animal groups by insulin tolerance test. The rate of glucose clearance in response to exogenous insulin (0.5 U/kg) was reduced in HFD mice in comparison with STD mice. Honey supplementation prevented the loss of insulin sensitivity induced by HFD, as showed by the area under the curve for the insulin tolerance test (Figure 2E,F). Accordingly, HOMA-IR, an index of insulin resistance, was significantly higher in HFD mice than STD- or HFD-H animal groups (Figure 2G). Overall, our results suggest that honey supplementation is able to prevent/mitigate the peripheral insulin resistance induced by HFD consumption.

### 3.3. Honey Ingestion Mitigates Neurodegeneration Induced by HFD 

**Figure 3:**
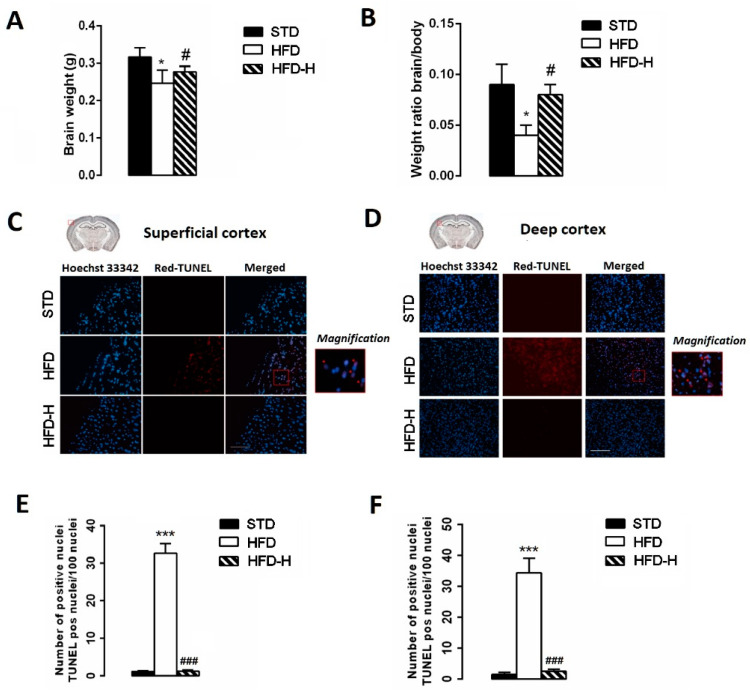
Impact of long-term honey ingestion on neuronal damage caused by HFD. Honey prevented neurodegeneration in HFD mice. (**A**) Brain weight; (**B**) Weight ratio brain/body; (**C**) TUNEL assay on superficial cerebral cortex sections; (**D**) TUNEL assay on deep cerebral cortex sections; (**E**) Number of apoptotic nuclei in superficial and deep (**F**) cerebral cortex in STD, HFD and HFD-H mice. Data are mean values ± S.E.M. (*n* = 8/group). * *p* < 0.05, *** *p* < 0.001 vs. STD; ^#^ *p* < 0.05, ^###^ *p* < 0.001 vs. HFD-fed mice. Microscope magnification 10×. Scale bar, 200 µm.

Whole-brain weight was determined at the sacrifice moment, and the brain/body weight ratio was calculated. The HFD brain mean weight was significantly lower than STD or HFD-H (Figure 3A) as well as the mean brain/body weight (Figure 3B). Moreover, we used the TUNEL assay in order to examine the number of apoptotic cells in superficial and deep cortical sections. We found a higher number of TUNEL-positive neurons in HFD mice than in STD- or HFD-H mice, suggesting a decrease in apoptotic cell death in the brain of HFD-H mice (Figure 3C–F).

**Figure 4:**
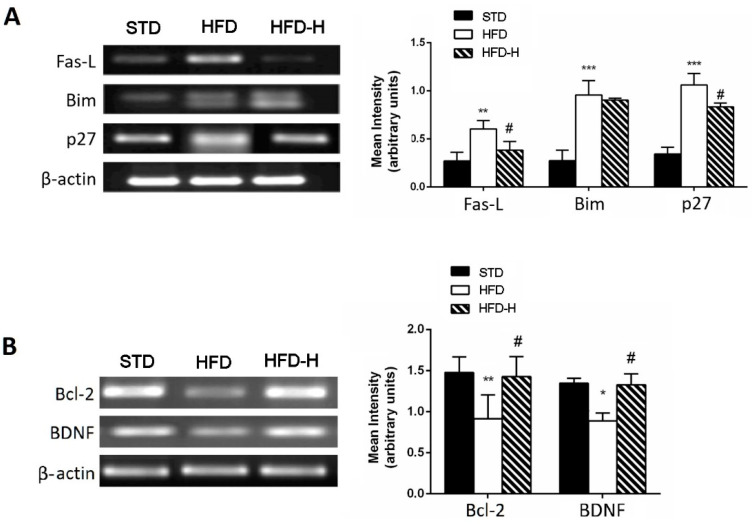
Effects of long-term honey ingestion on apoptosis-related gene expression in HFD mice. Honey downregulated pro-apoptotic gene expression and upregulated anti-apoptotic genes. (**A**) Representative images of the RT-PCR results and mRNA levels of pro-apoptotic genes: Fas-L, Bim and p27; (**B**) RT-PCR results and mRNA levels of survival genes: Bcl-2 and BDNF. Data are mean values ± S.E.M. (*n* = 8/group). * *p* < 0.05, ** *p* < 0.01, *** *p* < 0.001 vs. STD mice; ^#^ *p* < 0.05 vs. HFD. STD: standard mice; HFD: high fat diet mice; HFD-H: high fat diet supplemented with honey mice.

Honey supplementation was also able to prevent the increase in pro-apoptotic gene expressions, such as Fas-L and p-27, and the decrease in anti-apoptotic genes, such as Bcl-2 and BDNF caused by HFD, suggesting a neuroprotective effect of chronic honey ingestion (Figure 4A,B). 

### 3.4. Honey Ingestion Improves Insulin Signaling in Brains of HFD Mice

**Figure 5:**
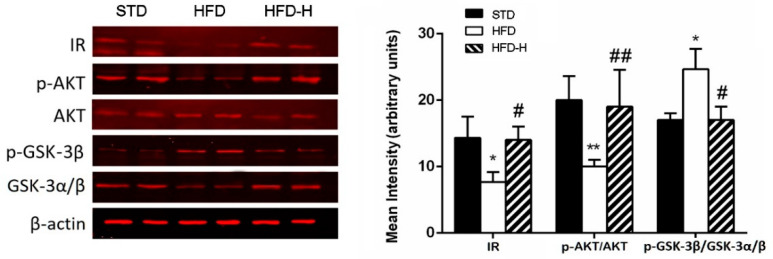
Effects of long-term honey ingestion on expression of insulin signaling protein HFD mice. Honey improved insulin signaling in HFD brain. Western blot of protein extracted from brain lysates of different mouse groups and incubated with anti-insulin receptor (IR), anti-p-AKT, anti-AKT, anti-p-GSK-3β, anti-GSK-3α/β and β-actin antibodies. Honey prevented the expression changes in insulin receptor (IR), p-AKT and p-GSK-3 caused by HFD. Data are mean values ± S.E.M. (*n* = 8/group). * *p* < 0.05, ** *p* < 0.01 vs. STD mice; ^#^ *p* < 0.05, ^##^ *p* < 0.01 vs. HFD. STD: standard mice; HFD: high fat diet mice; HFD-H: high fat diet supplemented with honey mice.

In order to verify if honey ingestion also improves central insulin resistance, we investigated the effects of HFD and honey supplementation on insulin signaling in the brain. Insulin receptor, AKT and GSK3-β expression were detected by Western blotting. In the brain, HFD feeding for 16 weeks decreased the expression of IR, p-Akt/Akt and upregulated GSK3-β/p-GSK3-β compared to controls. Honey supplementation prevented the down/upregulation of these alterations associated with HFD consumption (Figure 5), suggesting that chronic honey ingestion improves the impairment of insulin signaling in obesity conditions. 

In addition, we assessed expression changes in genes involved in central insulin resistance by using a Profiler PCR array. Twelve genes were up or downregulated by more than Two-fold in HFD-H fed mice compared to HFD. More specifically, we found an upregulation of adipokine Receptor (*adipoR1 and adipoR2*), insulin receptor (*InsR*) and its substrate (*Irs1),* and a downregulation of proinflammatory genes such as *Rbp4, Cd36 e Stat3* (Table 2).

### 3.5. Honey Supplementation Modifies Blood Levels of Leptin and Adiponectin

**Figure 6:**
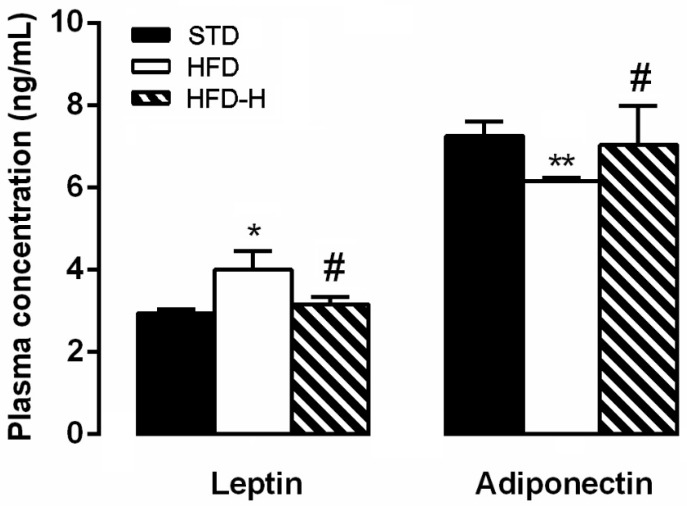
Effects of long-term honey ingestion on leptin and adiponectin concentration in HFD mice. Honey prevented the changes in plasma levels caused by HFD. Data are mean values ± S.E.M. (*n* = 8/group). * *p* < 0.05, ** *p* < 0.01 vs. STD mice; ^#^ *p* < 0.05 vs. HFD. STD: standard mice; HFD: high fat diet mice; HFD-H: high fat diet supplemented with honey mice.

Compared with the levels in the STD group, the HFD mice displayed significantly higher leptin and lower adiponectin plasma concentrations (Figure 6). Honey supplementation prevented the changes in the plasma levels of these hormones (Figure 6).

## 4. Discussion

The major findings from the present study suggest that honey ingestion effectively attenuates obesity-related peripheral and central insulin resistance. Moreover, honey ingestion prevents HFD-induced neurodegeneration by modulating positively brain gene expression involved in insulin signaling, neuroinflammation, apoptosis and some adipokine receptors. 

Nowadays, it is widely documented that obesity, type 2 diabetes or different metabolic disorders, including metabolic syndrome and NAFLD, increase the risk of cognitive decline, and these diseases have been linked to AD-type pathology [25,26,27,28,29]. In our study, we induced obesity in mice by HFD because this approach mimics the usual route of obesity occurrence in humans, causing positive energy balance and an increase in visceral fat [30]. In addition, in rodents, long-term HFD intake results in obese peripheral and central insulin resistance and impaired brain functions, as suggested by the presence of brain mitochondrial dysfunction oxidative stress, neuroinflammation, impaired synaptic plasticity and cognitive decline [10,15,31,32,33,34,35,36]. Therefore, an HFD obese mouse is a useful model for verifying the impact of functional food on obesity-related impairments, including neurodegeneration.

The results of the present study provide evidence for the beneficial effects of chronic honey ingestion in obese mice. In particular, dietary honey supplementation prevented the fasting hyperglycemia and the impairment of the glucose response during the glucose tolerance test, and it improved the plasma insulin concentration, insulin sensitivity and HOMA index, suggesting a preventive effect of the long-term honey ingestion in HFD-induced impairment of glucose homeostasis. On the other hand, honey hypoglycemic properties have been pointed out not only in diabetes rodent models but also in healthy subjects and diabetic patients [17,22,37,38]. In our experimental conditions, we did not observe any significant impact on weight gain or plasma lipid concentrations in HFD-H mice, ruling out an improvement of lipid metabolism as responsible for the observed effects on glucose metabolism. 

Moreover, our results obtained from Western blotting and microarray experiments suggest that alterations in glucose homeostasis induced by HFD consumption in mice are associated with impaired insulin signaling in the brain, and this effect is mitigated by honey supplementation. In fact, the expressions of p-AKT and p-GSK3 in HFD-H mice, which, respectively, decreased and increased in HFD mouse brain, index of central insulin resistance, were similar to STD animals. Moreover, the downregulation of insulin receptor and its substrate, Irs1, found in the brain of HFD mice, was not observed in HFD-H mice, supporting the hypothesis that regular intake of honey restored brain insulin signaling in HFD obese mice. To our knowledge, this is the first study to provide evidence for honey’s ability to improve brain insulin signaling. The capacity of regular honey consumption to improve brain insulin signaling could explain its protective role on neuronal function. In fact, insulin resistance has been reported to lead to impairment of neuronal homeostatic functions, oxidative stress, DNA damage, mitochondrial dysfunction, and, consequently, cell death [28,39]. Moreover, it is well accepted that impairment of insulin signaling leads to the downregulation of various neuroprotective genes [29]. Therefore, chronic ingestion of honey could protect the brain from HFD-caused damage by improving central insulin sensitivity. In fact, in our experimental model, HFD induced chronic neurodegeneration as suggested by the lower brain/body weight ratio and the higher number of apoptotic neurons in the HFD cortex than STD. However, honey supplementation attenuated these detrimental effects suggesting a neuroprotective effect of chronic honey ingestion. Moreover, previous studies on animals reported that intake of honey was beneficial and improved memory loss and cognitive decline caused by different conditions, such as aging, stress, ovariectomy [40,41,42] and neuroinflammation induced by Aβ-42 injection [43]. The beneficial effects of honey on the brain have been associated with the presence of components such as flavonoids and phenolic acids that can improve oxidative stress and oxidative stress-linked effects [17,44,45].

Although we did not take into account the oxidative state of the HFD-brain, our results clearly show that honey exerts neuroprotective effects on the HFD brain because it was able also to prevent the increase in pro-apoptotic gene expressions, such as Fas-L and p-27, and the HFD-caused decrease in anti-apoptotic genes and survival factors, such as Bcl-2 and BDNF. Moreover, the downregulation of inflammation-related genes, such as Cd36, Adgre1 or Acsl4 that promotes proinflammatory cytokine production from microglia and neuronal death [46], as revealed by our microarray experiments, supports, once more, the honey neuroprotective properties. A positive effect due to the honey ingestion could be represented by a reduction in retinol-binding protein 4 (RBP4) gene expression. In fact, RBP4 is an adipokine related to the dysregulation of energy metabolism, insulin resistance, diabetes mellitus and obesity [47], and it was reported to play a role also as an inflammatory neurotrophic adipokine [48]. In addition, suppression of signal transducer and activator of transcription 3 (STAT3) in the HFD-H brain would represent evidence for a positive impact of honey because STAT3 was related to different microglia-dependent proinflammatory responses [49,50,51]. 

Disturbed neuronal lipid and cholesterol homeostasis has been linked to central insulin resistance contributing to the pathogenesis of neurodegenerative diseases, such as Alzheimer’s disease (AD) [15,52,53]. In the current study, genes related to lipogeneses, such as CCAAT/enhancer-binding protein (Cebpa) and sterol regulatory element-binding factor 2 (Srebf2), were downregulated in the HFD-H brain in comparison with the HFD brain, suggesting a further mechanism by which honey supplementation can protect the cerebral neurons. 

Since dysregulated leptin and adiponectin secretions were reported to be one of the links among obesity, insulin resistance and neurodegenerative disorders [54], we measured plasma levels of these adipokines in the different animal groups. On the other hand, receptors for these adipokines, present in various brain regions, are involved in the pathogenesis of neurodegenerative diseases [54,55]. It is well accepted that in obesity, leptin resistance develops, leading to increased leptin production by adipose tissue and hyperleptinemia in an attempt to compensate for the low leptin responsiveness [56]. Therefore, leptin plasma levels are related positively to adiposity [57]. Leptin levels were lower in the HFD-H group than HFD mice, despite the fact that the fat mass was similar, suggesting the ability of honey to prevent also the leptin unbalance caused by HFD. Moreover, central leptin resistance mediated by downregulation of leptin receptors (Lepr) and/or by deficient leptin signaling downstream LepRs are involved in neuronal deficits [58]. Therefore, the brain upregulation of Lepr gene induced by honey ingestion could be considered as one of the mechanisms involved in honey neuroprotection. In our study, the plasma level changes in adiponectin caused by honey can be interpreted as an improvement tool of insulin resistance and the consequent neuronal impairment. In fact, adiponectin is reported to increase insulin sensitivity to have anti-inflammatory and antioxidant activity [58]. Indeed, reduced levels of circulating adiponectin are found in obesity, likely causing insulin resistance [59]. Moreover, adiponectin and adiponectin receptor (AdipoRs) agonists are known to be neuroprotective in primary rat hippocampal neurons and various rodent models of cerebral damage [58,60,61], and we found a higher expression of adipoRs in the brain of HFD-H mice in comparison with HFD mice.

## 5. Conclusions

The present study suggests that long-term ingestion of chestnut honey attenuates peripheral and central insulin resistance and prevents HFD-induced neurodegeneration by modulating positively brain gene expression involved in insulin signaling, neuroinflammation, apoptosis and some adipokine receptors. Our results support the functional action of chronic honey consumption that could be achieved considering a daily honey intake of about 10 g [62].


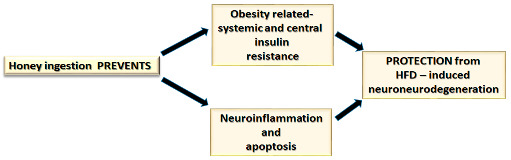


## Figures and Tables

**Table 1:** Oligonucleotide sequence of primers for RT-PCR.

Gene	Forward Primer	Reverse Primer	T° Annealing
*Fas-L*	5′-CAAGTCCAACTCAAGGTCCATGCC-3′	5′-AGAGAGAGCTCAGATACGTTTGAC-3′	58 °C
*Bim*	5′-AACCTTCTGATGTAAGTTCT-3′	5′-GTGATTGCCTTCAGGATTAC-3′	58 °C
*p27*	5′-TGCGAGTGTCTAACGGGAG-3′	5′-GTTTGACGTCTTCTGAGGCC-3′	59 °C
*Bcl-2*	5′-ATGTGTGTGGAGAGCGTCAA-3′	5′-AGAGACAGCCAGGAGAAATCA-3′	47 °C
*BDNF*	5′-GGCTGACACTTTTGAGCACGTC-3′	5′-CTCCAAAGGCACTTGACTGCTG-3′	52 °C
*IL-1β*	5′-TCATGGGATGATGATAACCTGCT-3′	5′-CCCATACTTTAGGAAGACACGATT-3′	50 °C
*IL-6*	5′-CTGGTGACAACCACGGCCTTCCCT-3′	5′-ATGCTTAGGCATAACGCACTAGGT-3′	54 °C
*TNF-α*	5′-AGCCCACGTCGTAGCAAACCA-3′	5′-GCAGGGGCTCTTGACGGCAG-3′	53 °C
*Β-actin*	5′-CGGGATCCCCGCCCTAGGCACCAGGGT-3′	5′-GGAATTCGGCTGGGGTGTTGAAGGTCTCAAA-3′	60 °C

**Table 2:** Expression profiles of genes in HFD-H/HFD which were significantly upregulated or downregulated by 2-folds.

Gene Name	Protein	HFD-H/HFD
Acsl4	Acyl-CoA synthetase long-chain family member 4	−2.30
Adgre1	Adhesion G Protein-Coupled Receptor E1	−2.12
Adipor1	Adiponectin receptor 1	2.47
Adipor2	Adiponectin receptor 2	2.32
Cd36	CD36 antigen	−3.33
Cebpa	CCAAT/enhancer binding protein (C/EBP), alpha	−2.83
Insr	Insulin receptor	3.77
Irs1	Insulin receptor substrate 1	4.17
Lepr	Leptin receptor	5.44
Rbp4	Retinol binding protein 4, plasma	−4.46
Srebf2	Sterol regulatory element binding factor 2	−2.12
Stat3	Signal transducer and activator of transcription 3	−2.39

## Supplementary Materials

The following supporting information can be downloaded at: https://www.mdpi.com/article/10.3390/nu14040756/s1. Table S1: Composition of the different diets. Table S2: Composition of honey.
