# Supplementary material for: Preventive Impact of Long-Term Ingestion of Chestnut Honey on Glucose Disorders and Neurodegeneration in Obese Mice
Source: Nutrients. 2022 Feb 11;14(4):756. doi: 10.3390/nu14040756 (PMC8879402; doi:10.3390/nu14040756)
Supplement: Supplementary file 1 [file nutrients-14-00756-s001.zip › table S1 diets composition.pdf]

Table S1. Composition and energy densities of STD and HFD .

Ingredient (g/kg)	STD	HFD
Acid Casein 741	200	265.00
L-Cystine	2.8	4
Maltodextrine-0032	33.2	160
Sucrose	300	90
Cellulose (Arbocel)	50	65.5
Soybean oil	25	30
Lard	19	220
Vitamin mix AIN-93-VX-PF2439	10	21
Mineral mix AIN-93G-MX-PF2348	45	48
Choline bitartrate	1.9	3
Calcium Phosphate dibasic	13	3.4
Pistachio	-	-
Total Energy, Kcal/g	3.5	6
Protein, %	20	20
Carbohydrate, %	70	20
Fat, %	10	60

Abbreviations are: STD, Standard diet. HFD, high fat diet. This study used 4RF25, PF4051/D and PF4215/C-R&S34/16 diets (Mucedola s.r.l.) as STD and HFD respectively. Composition of these diets is from the Mucedola Web site.
