# Supplementary material for: Preventive Impact of Long-Term Ingestion of Chestnut Honey on Glucose Disorders and Neurodegeneration in Obese Mice
Source: Nutrients. 2022 Feb 11;14(4):756. doi: 10.3390/nu14040756 (PMC8879402; doi:10.3390/nu14040756)
Supplement: Supplementary file 1 [file nutrients-14-00756-s001.zip › table S2 honey composition.pdf]

Supplementary data

Table S2. Composition of Sicilian black bee chestnut honey

Honey Chestnut Components	g/100g
D(+)Maltose	2.44
Glucose	21.00
D(-)-Fructose	33.00
D-Threalose	1.03
D-Apiose	3.35
Sucrose	1.53
**Phenolic compounds**	µg/100g
Apigenin	378.7
Kaempferol	7545
Quercetin	7184
Rutin	71.02
Myricetin	1871
Hesperidin	17.07
Naringenin	103.2
Pinocembrin	1549
Caffeic acid	416.2
Ferulic acid	66.38
Gallic acid	85.66
Syringic acid	9.97
Vanillic acid	230.0
Catechin	nd
Epicatechin	nd
Apigenin	378.7
Kaempferol	7545
Quercetin	7184
